# Mechanism of Social Stress-Related Erectile Dysfunction in Mice: Impaired Parasympathetic Neurotransmission and Ketamine

**DOI:** 10.3390/ijms241511973

**Published:** 2023-07-26

**Authors:** Shu-Yu Wu, Tze-Chen Chao, Chun-Kai Hsu, His-Hsien Chang, Stephen Shei-Dei Yang

**Affiliations:** 1 Department of Urology, Taipei Tzu Chi Hospital, Buddhist Tzu Chi Medical Foundation, New Taipei 23142, Taiwan; nobookrain2014@gmail.com (S.-Y.W.); s99311106@gmail.com (T.-C.C.); svevi0614@gmail.com (C.-K.H.); changhh@livemail.tw (H.-H.C.); 2 Department of Urology, School of Medicine, Tzu Chi University, Hualien 97004, Taiwan

**Keywords:** erectile dysfunction, social stress, ketamine, sexual dysfunction

## Abstract

This study aimed to investigate the mechanism underlying social stress (SS)-induced erectile dysfunction (ED) and evaluate the effects of a single subanesthetic dose of ketamine on SS-related ED. Male FVB mice were exposed to retired male C57BL/6 mice for 60 min daily over a 4-week period. In the third week, these FVB mice received intraperitoneal injections of either saline (SSS group) or ketamine (SSK group). Erectile function was assessed by measuring the intracavernosal pressure (ICP) during electrical stimulation of the major pelvic ganglia. Corpus cavernosum (CC) strips were utilized for wire myography to assess their reactivity. Both SSS and SSK mice exhibited significantly lower ICP in response to electrical stimulation than control mice. SS mice showed increased contractility of the CC induced by phenylephrine. Acetylcholine-induced relaxation was significantly reduced in SSS and SSK mice. Sodium nitroprusside-induced relaxation was higher in SSS mice compared to control and SSK mice. Nicotine-induced neurogenic and nitric oxide-dependent relaxation was significantly impaired in both SSS and SSK mice. An immunohistochemical analysis revealed co-localization of tyrosine hydroxylase and neuronal nitric oxide synthase-immunoreactive fibers in the CC. These findings highlight the complex nature of SS-related ED and suggest the limited efficacy of ketamine as a therapeutic intervention.

## 1. Introduction

Previous studies have shown that prolonged exposure to social stress (SS) induces lower urinary tract dysfunction (LUTD) [1,2,3], resulting in urinary retention and abnormal urodynamics [2,4], as central parasympathetic neurotransmitters may be decreased by SS, therefore reducing detrusor contraction [2,4]. SS also causes copulatory disorder [5,6,7] and depression secondary to neurovascular pathology [8]. Previous studies have shown that reduced nitrogen oxide (NO) is correlated with depression and anxiety in humans [9,10]. Preclinical research has repeatedly demonstrated that effects resembling those of antidepressants can be achieved by suppressing NO production, such as direct suppression of neuronal nitric oxide synthase (nNOS) and/or inducible nitric oxide synthase (iNOS), where NOS is a family of enzymes catalyzing the production of NO from L-arginine [11]. However, the complexity of the neurobiology of depression and the multifaceted nature of NO signaling makes it challenging to draw definitive conclusions. In other studies, nNOS-derived NO-caused vasorelaxation has been reported, with reduced relaxation in the cerebral arteries of adult spontaneously hypertensive rats (SHRs) [12]. Lee and Saito reported that adventitial parasympathetic cholinergic nerve terminals were decreased in all cerebral arteries of SHRs [13], therefore implying that the sympathetic–parasympathetic nerve interaction (or axo–axonal interaction) mechanism results in neurogenic relaxation in the cerebral basilar arteries and is impaired by the decrease in parasympathetic cholinergic nerve terminals in adult SHRs.

During penile erection, NO is an important neurotransmitter in the principal pathway [14,15]. Previous animal studies have shown that NO released from endothelial cells in the corpus cavernosum (CC) of mice will inhibit contractions [16]. In unilateral cavernosal denervation rats, the intracavernous pressure (ICP) and NOS fibers were observed to be reduced in the CC of these rats [15]. A decrease in nNOS was observed to be accompanied by nerve degeneration in penile tissue in erectile dysfunction (ED) patients [17]. However, the impact of the peripheral parasympathetic nitrergic nerve in SS-induced ED remains unclear.

Ketamine is an antagonist of the *N*-methyl-D-aspartic acid (NMDA, an excitatory amino acid) receptor complex [18,19], and a single infusion of a subanesthetic dose produces a rapid and long-lasting antidepressant effect [20], suggesting that ketamine may be useful for protecting against stress-induced disorders [21]. Brain-derived neurotrophic factor in the prefrontal cortex may be related to the long-lasting antidepressant effect of ketamine, as seen in the social defeat stress animal model [22]. Long-term use of ketamine could induce both LUTD and ED in humans in a dose- and duration-related model [23,24]. We previously reported the crucial role of the interaction between sympathetic and parasympathetic nerve terminals in the relaxation of the CC. This interaction is mediated by both nicotinic acetylcholine receptors (nAChR) and NMDA receptors. In addition, we observed that ketamine directly targets the major pelvic ganglia (MPG) in vivo, leading to the inhibition of cavernosal nerve neurotransmission. Furthermore, our in vitro experiments demonstrated that ketamine impairs the ability of nicotine to reduce relaxation in the CC [25]. Notably, our investigations involving chronic ketamine use did not yield positive results with respect to reversing LUTD in mice subjected to SS [26]. Therefore, the objective of this study was two-fold: firstly, to delve into the mechanism of the axo–axonal interaction in SS-related ED, and secondly, to investigate the effects of a single subanesthetic dose of ketamine on SS-related ED.

## 2. Results

### 2.1. Effects of SS on Voiding

**Figure 1:**
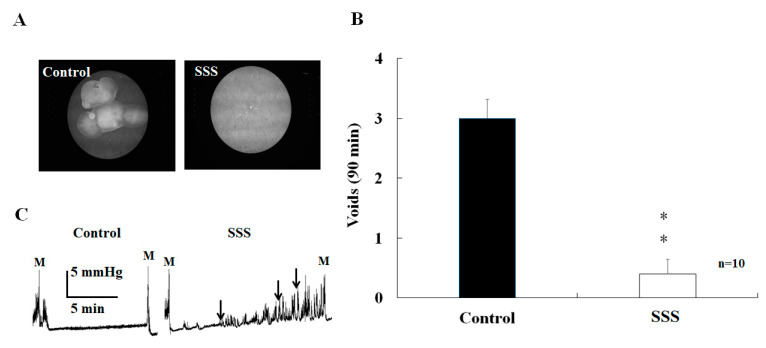
The mice exposed to the 4-week SS demonstrated a decreased number of urine spots on the filter paper (**A**) and the voiding frequency of SSS was statistically lower than that of control mice (**B**). The different cystometric detrusor contractions are shown in (**C**). ** *p* < 0.01.

Figure 1A depicts urine spot expression of control and SSS (SS mice, saline injection group) mice. It is inferred from Figure 1A that most SSS mice were unable to void, as seen from the little urine spots. The voiding frequency of SSS mice in a fixed period of 90 min was also significantly lower than that of the control mice (0.4 ± 0.24 vs. 3 ± 0.31, *p* < 0.01, Figure 1B). Cystometry recordings illustrated unstable detrusor contractions (black arrows) in SSS mice, which were not present in the control mice (Figure 1C).

### 2.2. Effects of SS on Urinary Bladder Pressure and ICP

**Figure 2:**
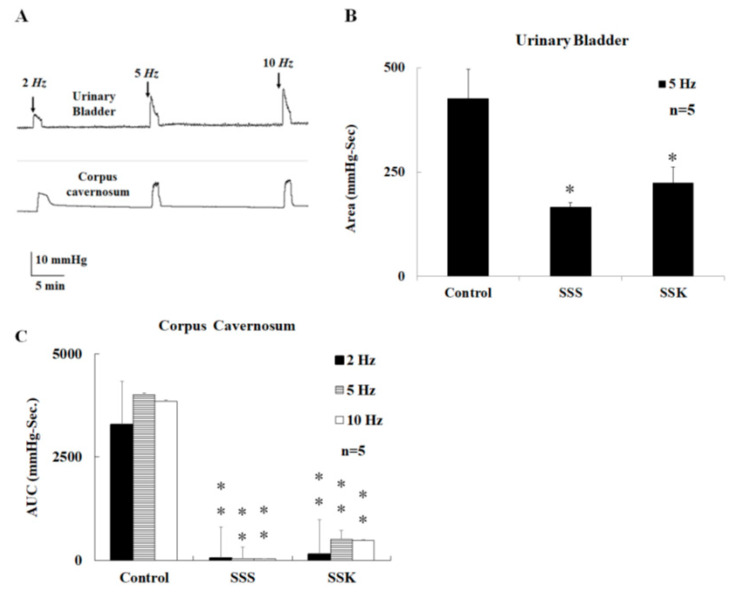
A representative tracing depicting the activation of MPG by electrical stimulation (2, 5, and 10 Hz), which caused urinary bladder contraction and an increase in the ICP in male mice (**A**). The electrical-stimulation-induced contraction of the urinary bladder was significantly greater in the control mice than in the SSS and SSK mice (**B**). The electrical-stimulation-induced elevation of the ICP was significantly greater in the control mice than in the SSS and SSK mice (**C**). * *p* < 0.05; ** *p* < 0.01.

The area under the curve (AUC) for the urinary bladder pressure of control mice was induced by electrical stimulation of the cavernous nerve and MPG (2, 5, and 10 Hz, Figure 2A). Both SSS and SSK (SS mice, ketamine injection group) mice demonstrated a lower AUC than that of control mice at 5 Hz (Figure 2B), with no statistical difference observed between the SSS and SSK mice (Figure 2B).

Both SSS and SSK mice demonstrated a lower AUC for the ICP and urinary bladder pressure than those of the control mice (Figure 2C), with no statistical difference noted between the SSS and SSK mice (Figure 2C).

### 2.3. Effects of SS on the Relaxation of Corpus Cavernosum Strips

**Figure 3:**
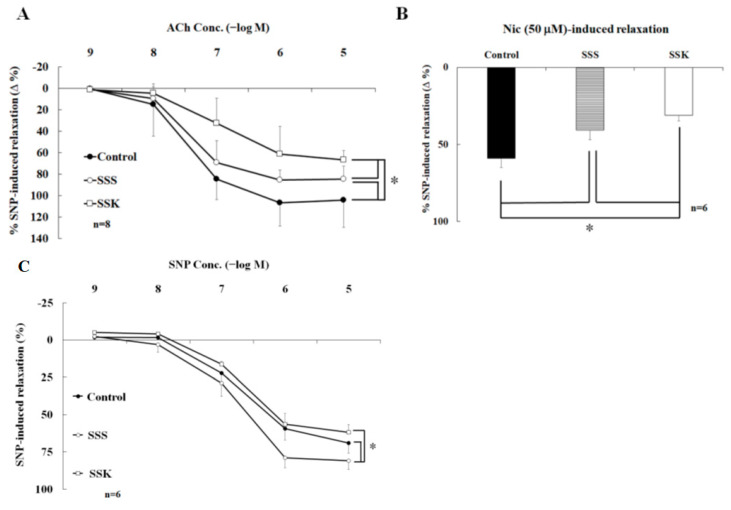
Effect of social stress on the NO-dependent relaxation of the smooth muscle of the corpus cavernosum. (**A**) ACh-induced relaxation; (**B**) nicotine-induced relaxation; (**C**) SNP-induced relaxation. * *p* < 0.05.

In this study, SS mice received intraperitoneal injections of either saline (SSS group) or ketamine (SSK group). In the presence of active muscle tone induced by phenylephrine (10 µM, a selective α1-adrenergic receptor agonist), the CC strips relaxed with applications of acetylcholine (Ach) in a concentration-dependent manner (10^−9^~10^−5^ M). Figure 3 shows the effect of SS on NO-dependent relaxation on the smooth muscle of the CC. The extent of relaxation of the CC strips of the SSS and SSK mice was significantly lower than that for the control mice (Figure 3A), with a statistical difference observed between the SSS and SSK mice (Figure 3A). Similarly, the extent of the relaxation of the CC strips after the application of nicotine (50 µM) was significantly lower in the SSS and SSK mice than in the control mice (Figure 3B). As for the extent of the relaxation of the CC strips induced by sodium nitroprusside (SNP, a NO donor) in a concentration-dependent (10^−9^~10^−5^ M) manner, the relaxation was significantly enhanced in the SSS mice compared with the control mice (Figure 3C), with no statistical difference observed between the SSK and control mice (Figure 3C).

### 2.4. Effects of Lidocaine on Nicotine-Induced Relaxation

**Figure 4:**
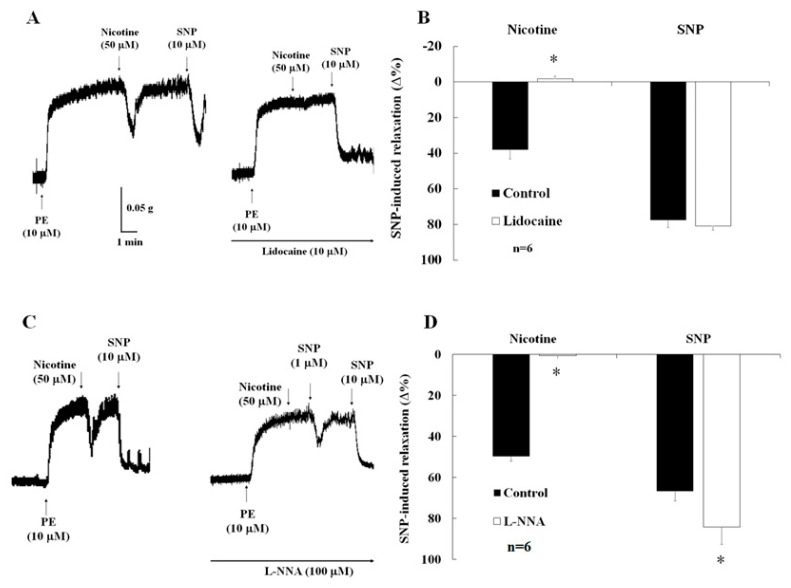
Effects of lidocaine and the NOS inhibitor on nicotine-induced relaxation in the control mice. (**A**,**B**) Nicotine-induced relaxation was abolished by lidocaine; SNP-induced relaxation was not inhibited by lidocaine; (**C**,**D**) nicotine-induced relaxation was abolished by Nomega-Nitro-L-arginine (L-NNA); SNP-induced relaxation was enhanced by L-NNA. * *p* < 0.05.

Figure 4 shows the effects of lidocaine and the NOS inhibitor on nicotine-induced relaxation in the control mice. In the presence of active muscle tone due to phenylephrine (10 µM), relaxation of the CC strips was observed with nicotine (50 µM). However, this relaxation was significantly reversed by lidocaine (10 µM, Figure 4A,B, 39.8 ± 5.0% vs. 0.6 ± 2.7%, *p* < 0.05). In contrast, the relaxation of the CC strips induced by SNP (0.1 µM) was not inhibited by lidocaine (10 µM, 75.5 ± 4.1% vs. 80.9 ± 1.8%, *p* > 0.05, Figure 4A,B).

### 2.5. Effects of NO Synthase Inhibitor on Nicotine-Induced Relaxation and ICP

In the presence of active muscle tone induced by phenylephrine (10 µM), relaxation of the CC strips was observed with nicotine (50 µM). This relaxation was significantly reversed by L-NNA (Figure 4C,D, 49.8 ± 2.3 vs. 0.6 ± 1.9, *p* < 0.05). In contrast, the relaxation of the CC strips induced by SNP (0.1 µM) was significantly enhanced by L-NNA (Figure 4D, 66.7 ± 4.7 vs. 84.3 ± 8.3, *p* < 0.05).

### 2.6. Effects of SS on the Contraction of Corpus Cavernosum Strips

**Figure 5:**
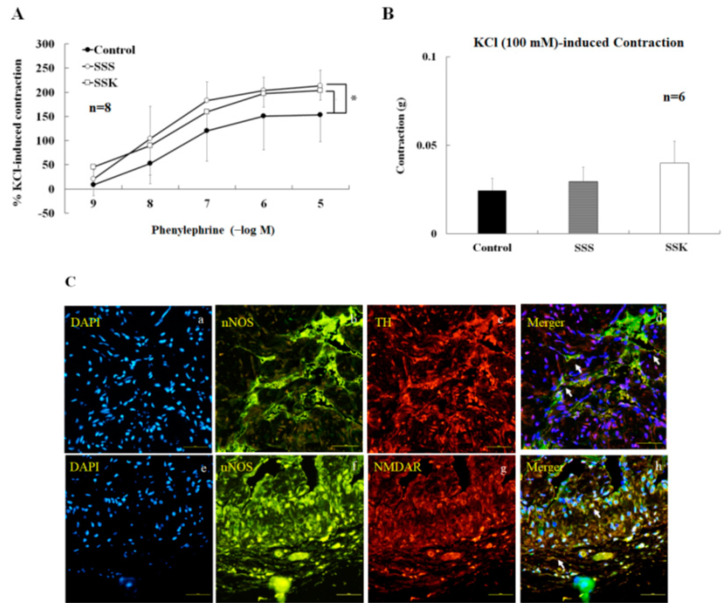
In the absence of active muscle tone, CC contracted upon the application of phenylephrine (10^−9^~10^−5^ M) in a concentration–response manner. The phenylephrine-induced contraction was significantly greater in the SSS and SSK mice than in the control mice (n = 6, * *p* < 0.05), (**A**). KCl (100 mM)-induced maximal CC contraction did not show statistical differences between the three groups (n = 6, * *p* > 0.05 vs. control), (**B**). (**C**) The association DAPI (blue, **a**), nNOS- (green, **b**), and TH-immunoreactive fibers (red, **c**), and double-labeled (**d**) were observed in the CC of the FVB mice. In addition, the association DAPI (blue, **e**), nNOS-immunoreactive fibers (green, **f**), and NMDAR (red, **g**), and double-labeled (**h**) in the corpus cavernosum of the FVB mice are presented for comparison. The scale represents 100 μm.

In the absence of active muscle tone, the contraction of CC strips was induced by phenylephrine in a concentration-dependent manner (10^−9^~10^−5^ M). The contractility of the CC strips of the SSS and SSK mice was significantly higher than that of the control mice (Figure 5A, *p* < 0.05). No statistical difference was observed for the maximal contraction induced by KCl (100 mM) among the three groups (Figure 5B, *p* > 0.05). Freshly dissected CC preparations were fixed using a formalin solution, and observations of the association between tyrosine hydroxylase (TH, a sympathetic nerve fiber marker)- and nNOS-immunoreactive fibers as well as the presence of double-labeled TH/nNOS fibers (Figure 5C(a–d)) in the CC of the FVB mice were conducted. In addition, nNOS -immunoreactive fibers and their association with NMDA receptors (NMDAR), along with the presence of double-labeled nNOS/NMDAR fibers (Figure 5C(e–h)), were also observed in the CC of the FVB mice. Notably, these TH/nNOS-immunoreactive fibers and NMDAR/nNOS-immunoreactive fibers exhibited co-localization within the same nerve fibers (Figure 5C).

## 3. Discussion

This study demonstrates that SS (SSS group) impairs neurogenic and endothelial NO-dependent relaxation of the CC and leads to ED, which is not improved by a single subanesthetic dose of ketamine (SSK group) and, instead, further exacerbates ED. The SS mice had a statistically lower voiding frequency and a smaller voiding volume than those of the control mice (Figure 1). These findings concur with previous studies [1,2,3,26]. Under electrical stimulation, the SS mice had lower ICP values than the control mice, and this was not reversed with single-dose subanesthetic ketamine administration (Figure 2C). These results suggest that SS may impair peripheral parasympathetic transmission, detrusor contractility, and erectile function. It has been reported that electrical stimulation of the MPG causes bladder contractions [27,28]. Our results show that electrical stimulation can cause both bladder and CC contraction. AUC is a composite variable, incorporating frequency, amplitude, and duration, and has been found to be a useful composite measure of involuntary bladder activity during the comparison of dose–response relationships [29,30]. Our results suggest that electrical stimulation induced an increase in MPG activity and was accompanied by an increase in intravesical pressure.

SS-related ED may be associated with a reduction in NO inside the CC and exogenous NO-enhanced CC relaxation of the SSS mice (Figure 3C), possibly as a compensatory mechanism. SNP-induced CC relaxation was enhanced by SS in a concentration-dependent manner, but it was reversed by a subanesthetic dose of ketamine (Figure 3A). It is a known fact that norepinephrine (NE) can induce CC contractions, while ACh can induce endothelial-dependent relaxation [31,32,33]. In this study, Ach induced concentration-dependent and endothelial NO-dependent relaxation, which was decreased in the SS mice, and single-dose ketamine administration further decreased ACh-induced relaxation in the CC (Figure 3B). It was previously reported that ketamine can decrease endothelial NOS activity in the blood vessels due to a reduction in intracellular calcium levels [34] and a reduction in the intracellular Ca^2+^ concentration in vascular smooth muscle cells [35]. Therefore, ketamine may decrease intracellular calcium levels of endothelial cells in the CC.

In the control mice, nicotine-induced CC relaxation was inhibited by lidocaine (Figure 4A,B) and L-NNA (Figure 4C,D), which suggests that nicotine induces neurogenic NO-dependent relaxation in the CC. In neurophysiology, voltage-dependent ion channels play an important role in the generation and propagation of nerve impulses [36]. The blockade of the Na^+^ channels induced by ketamine is an important feature of local anesthetic action [37]. In the central neuron system, ketamine causes a decrease in brainstem parasympathetic cardiac neuron activity [38]. In addition to the central neuron system, it was reported that nicotine acts on the nAChRs located on the peripheral parasympathetic nitrergic nerves, thereby evoking the release of NO from these nerve terminals and inducing a relaxation response in the CC of rabbits [39]. The α7-nAChR expressed in the CC of rats modulates the nicotine-related neurogenic relaxation response [40]. SS decreases nicotine-induced CC relaxation due to damage to parasympathetic nitrergic nerves, while ketamine may not reverse this change. Therefore, our results suggest that the neurogenic- and endothelial-dependent relaxation are reduced by SS and further decreased by ketamine administration in the CC.

KCl-induced maximal CC contraction was not statistically different among the three groups, but phenylephrine-induced CC contraction was enhanced in the SSS and SSK mice (Figure 5A,B). These results suggest that the contractility of the smooth muscle of the CC was not different among the study groups. The present findings for the control mice are consistent with previous observations that maximal contraction due to phenylephrine and the relaxation response due to nitroprusside are increased in the CC in both the endothelium NOS and in nNOS knockout mice [41]. Meanwhile, SNP-induced relaxation was enhanced by pretreated L-NNA in this study. This result further provides evidence that NO-dependent relaxation was impaired by SS and ketamine in the CC.

Our recent results show that NMDAR plays an essential role in the sympathetic–parasympathetic nerve interaction transmission pathway [25]. In Figure 5C, the TH-/nNOS-immunoreactive fibers and NMDAR/nNOS-immunoreactive fibers appear to be co-localized in the same nerve fibers. In this study, nicotine was shown to be capable of inducing relaxation of the CC. Nicotine plays a role at the sympathetic nerve terminals, causing NE release and acting on the beta-adrenergic receptors (β-ARs) on the parasympathetic/cholinergic nerve terminals. The stimulation of β-ARs may promote the action of NMDAR, thereby enhancing the conversion of L-arginine to NO by nNOS. Furthermore, this axo–axonal interaction mechanism is dependent on intact sympathetic nerves. MK-801 and ketamine inhibit nicotine-induced relaxation of the CC, therefore affecting NMDARs.

**Figure 6:**
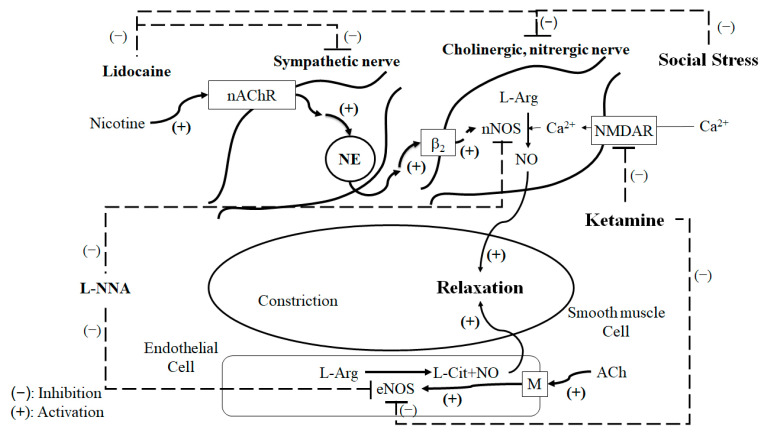
Overview of the proposed mechanism of SS-related ED based on the results.

Figure 6 summarizes the proposed mechanism of SS-related ED and ketamine based on the results through a schematic illustration. Nicotine activates nicotinic acetylcholine receptors of the sympathetic nerves and facilitates the release of NE inside sympathetic nerves. Subsequently, NE-stimulated adrenergic receptors (β-ARs) act on the parasympathetic/cholinergic nerve, leading to the production of NO, thereby causing relaxation of the CC. This study is a continuation of previous experiments. Our previous study demonstrated that nicotine induces NO-dependent neurogenic relaxation in the CC. This relaxation is dependent on the interaction of the sympathetic and parasympathetic nerves (axo–axonal interaction), which may be mediated by the NMDA receptor [25]. In this study, our results show that tyrosine hydroxylase (TH)-/nNOS-immunoreactive fibers are localized in the CC, and nicotine-induced relaxation is inhibited by Nω-nitro-L-arginine and lidocaine. These results are consistent and further support our conclusion.

## 4. Materials and Methods

### 4.1. SS Model

Here, we detailed our experimental procedure involving FVB and C57BL/6 mice, which was approved by the Laboratory Animal Care and Use Committee of our institute (109-IACUC-006). Five-week old male FVB mice were subjected to a controlled light (12 h light/dark cycles from 7:00 to 19:00) and constant temperature (21 to 23 °C) environment. The FVB mice were randomly assigned to a group exposed to SS for 60 min for seven consecutive days for 4 weeks or a control group not exposed to SS. During each SS exposure, an intruder FVB mouse was introduced into the cage area of an unfamiliar and aggressive C57BL/6 resident mouse. A typical SS exposure resulted in intruder subordination and was operationally defined when the FVB mice intruder adopted a supine posture. Following defeat, wire mesh partitions were placed in the cage to prevent direct physical contact between the resident and intruder. At the same time, other continuous contacts (including visual, olfactory, and auditory) were permitted for 60 min during each SS exposure. On the other hand, control mice were placed in a separate novel cage behind a wire partition for 60 min every day without any SS exposure. After each training session, the experimental and control mice were returned to their respective cages [26].

### 4.2. Ketamine Administration Procedures

The FVB mice were subjected to the SS protocol for three weeks, and then the mice were stressed for another week before further randomization into two groups: the SSS group, which received a single intraperitoneal injection of normal saline, and the SSK group, which received a single dose intraperitoneal injection of ketamine. The dose of ketamine was 25 mg/kg (United Biomedical, Inc., Hsinchu, Taiwan).

### 4.3. Urinary Bladder Pressure

In vivo urinary bladder pressure was measured at the end of the 4-week SS protocol. Upon completing the SS exposure, subjects were anesthetized with urethane (500 mg/kg) and chloralose (50 mg/kg). After lower abdominal wall incision and bladder identification, a saline-filled polyethylene (PE) 10 catheter with a blunt end was sutured to the bladder dome. After determining the optimal length, the PE10 catheter was secured with 6-0 sutures. The muscle and skin layers were sutured separately using nonabsorbable sutures. The bladder pressure was recorded with an MP36 polygraph (Biopac Systems Inc., Santa Barbara, CA, USA) and Biopic Student Lab (BSL) 3.7.3 software (Biopac Systems Inc., Santa Barbara, CA, USA) at a reasonable filling rate (1.8 mL/h).

### 4.4. ICP Measure

After 4 weeks of SS, the mice were subjected to in vivo ICP measurement. The mice were anesthetized with urethane (500 mg/kg) and chloralose (50 mg/kg). The abdominal wall was incised and dissected until the urinary bladder was reached, with careful removal of the surrounding tissues to avoid bleeding. The MPG was exposed, and the electrode wire was carefully passed through to hook the cavernosal nerve and the MPG, with the other end connected to an electrical stimulator (Grass, SD9J) [26]. The PE10 catheter was filled with normal saline containing heparin (100 IU) with one end connected to a 30-gauge needle, which was carefully pierced into the CC of the penis, and the other end connected to a port of the pressure sensor. The ICP was recorded on an MP36 polygraph (Biopac Systems Inc., Santa Barbara, CA, USA) and Biopac Student Lab (BSL) 3.7.3 software (Biopac Systems Inc., Santa Barbara, CA, USA). The cavernous nerve and MPG were then electrically stimulated (ES, 2–10 Hz, 50 msec, and 2.5 V) over 60 s, while the ICP was measured and the total ICP was monitored by calculating the AUC from the start of cavernous nerve stimulation to its return to the baseline.

### 4.5. Tissue Preparation

The FVB mice were sacrificed by cervical dislocation after anesthesia with urethane (500 mg/kg, ip) and chloralose (50 mg/kg, ip). The CC was dissected and placed in 4 °C oxygenated (95% O_2_ and 5% CO_2_) Krebs’ solution. The composition of the Krebs’ bicarbonate solution (in mM) was as follows: NaCl 117, NaHCO_3_ 25, KCl 4.7, CaCl_2_ 2.5, MgSO_4_ 1.2, KH_2_PO_4_ 1.2, glucose 11.1, and calcium disodium ethylenediamine tetraacetate (EDTA) 0.023.

### 4.6. Tissue Bath Wire Myography

The dissected and well-prepared CC tissues were evaluated under a dissecting microscope. They were then mounted on a stainless-steel rod and a platinum wire in a tissue bath containing 20 mL of Krebs’ solution. The Krebs’ solution was equilibrated with a mixture of 95% oxygen and 5% carbon dioxide and maintained at a temperature of 37 °C. Tension changes in the CC strips were measured using an isometric transducer (FT03C; Grass). The CC strips were allowed to equilibrate in the Krebs’ solution for 60 min and were mechanically stretched to achieve a resting tension of 0.2 gm. The experimental procedure consisted of the following steps:

Step 1: After the equilibration period, the resting muscle tone of the CC strips was measured by applying increasing concentrations of phenylephrine (ranging from 10^−9^ to 10^−5^ M).

Step 2: The CC strips were precontracted using a concentration of phenylephrine (10 µM), and then the relaxation effects were induced by applying ACh (ranging from 10^−9^ to 10^−5^ M) and nicotine (50 µM) in succession. These relaxation responses were recorded using an MP36 polygraph and Biopac Student Lab (BSL) 3.7.3 software.

Between Step 1 and Step 2, there was a 45-min washout period with Krebs’ solution to remove any residual effects of phenylephrine.

Step 3: The maximal contraction of the CC strip was induced by applying KCl at a concentration of 100 mM.

It is important to note that only one isolated CC strip per animal was used in the myography study. The extent of smooth muscle contraction induced by phenylephrine is expressed as a percentage of the maximum contraction induced by KCl (100 mM). Similarly, the extent of smooth muscle relaxation induced by ACh and nicotine is expressed as a percentage of the maximum relaxation induced by SNP at a concentration of 0.1 mM.

### 4.7. Double-Labeled Immunohistochemistry

Fresh CCs were dissected and placed in formalin solution (neutral buffered, 10%) at room temperature. After five washes in PBS (pH 7.4), the CCs were permeabilized and nonspecific sites were blocked with 2% bovine serum albumin in 0.25% Triton X-100-PBS for 30 min at room temperature. The CCs were incubated with the following primary antibodies at 4 °C for 24 h: rabbit anti-nNOS (1:500), combined mouse anti-NMDAR (1:500) or rabbit anti-nNOS (1:500), and combined mouse anti-tyrosine hydroxylase (TH, 1:500). After rinsing with PBS (pH 8.2) thrice, the CCs were incubated with the secondary antibodies for 1 h at room temperature. The secondary antibodies were FITC-labeled rabbit anti-rabbit IgG (1:500) and rhodamine-labeled mouse anti-mouse IgG (1:1000). The sections were incubated with 4′,6-diamidino-2-pheny-lindole (DAPI) for nuclear staining after washing out the secondary antibodies. After another PBS (pH 8.2) rinse, each CC was whole-mounted with Vectashield mounting medium on Vectabond-coated slides (Vector Laboratories, Burlingame, CA, USA). The final step was to use a fitted fluorescence microscope (Nikon E800 microscope, Nikon, Tokyo, Japan) to observe and record the processed specimens.

### 4.8. Drugs Used and Statistical Analysis

The following chemicals were used: ACh, CaCl_2_, EDTA, Glucose, HCO_3_, KCl, NaCl, NaMgCl_2_, NaH_2_PO_4_, nicotine, phenylephrine, SNP, rabbit anti-nNOS, mouse anti-NMDAR, mouse anti-tyrosine hydroxylase, FITC-labeled rabbit anti-rabbit IgG (1:500), rhodamine-labeled mouse anti-mouse, and DAPI (all from Sigma-Aldrich, St. Louis, MO, USA). The statistical analysis was performed using the program SigmaStat 3.5. A paired *t*-test was used to compare differences in the same strip. Comparisons among multiple groups were made by the Bonferroni post hoc test following one-way or two-way ANOVAs. Comparisons between two groups were made with unpaired two-tail Student’s *t* tests. All values are presented as the mean ± SEM. A value of *p* < 0.05 was considered statistically significant.

## 5. Conclusions

In conclusion, our findings suggest that neurogenic- and endothelial-dependent relaxation of the CC is impaired by SS. Meanwhile, SS enhances phenylephrine-induced contraction. These results suggest that SS-related ED is associated with a decrease in nNOS in the CC. However, single-dose treatment of ketamine does not possess the ability to reverse SS-related ED. In addition, TH/nNOS immunoreactive fibers and nNOS/NMDAR immunoreactive fibers were found to be co-localized in the CC, which is consistent with and further supports our previous study, which showed that axo–axonal interaction in the CC and ketamine action on the NMDAR impair relaxation of the CC. These results emphasize the intricate nature of SS-related ED and the limited effectiveness of ketamine as a potential therapeutic intervention for this condition. Further research is required to explore alternative strategies for managing SS-related ED.

## Data Availability

Data are available upon request.
